# Restin suppressed epithelial-mesenchymal transition and tumor metastasis in breast cancer cells through upregulating mir-200a/b expression via association with p73

**DOI:** 10.1186/s12943-015-0370-9

**Published:** 2015-05-14

**Authors:** Zhenduo Lu, Dechuang Jiao, Jianghua Qiao, Sen Yang, Min Yan, Shude Cui, Zhenzhen Liu

**Affiliations:** Department of Breast surgery, Breast Cancer Center, Affiliated Cancer Hospital of Zhengzhou University, Henan Cancer Hospital, #127 Dongming Road, Zhengzhou, Henan 450008 People’s Republic of China; Department of Pathogen Biology, Basic Medical College of Zhengzhou University, #100 Science Road, Zhengzhou, 450001 People’s Republic of China

**Keywords:** Restin, EMT, Tumor metastasis, mir-200a/b, p73, Breast cancer, MAGE superfamily

## Abstract

**Background:**

Restin belongs to MAGE superfamily and is known as MAGE H1. Restin was firstly cloned from HL-60 cells treated with all-trans retinoic acid (ATRA). Previous studies showed a pro-apoptotic role of Restin in several cell lines. However, little information is available on its expression patterns and functions *in vivo*. Our study was performed to detect if Restin plays a role in breast cancer cells *in vitro* and *in vivo*.

**Methods and results:**

Real-time PCR and western blot were conducted to detect Restin expression in multiple breast cancer cell lines and Restin level was negatively related with cell motility. Restin overexpression and knockdown stable cell lines were established by transducing lentivirus into MCF-7 and MDA-MB-231 cells. Cell morphology, wound closure assay, transwell migration and invasion assays were performed to detect if Restin inhibited EMT. Our data showed that Restin overexpressed cells exhibited classical epithelial cell morphology, and Restin overexpression resulted in activation of epithelial markers and suppression of mesenchymal markers, and inhibition of cell migration and invasion. Tumor xenograft model was used to characterize the biological functions of Restin *in vivo*. We found that Restin overexpression led to reduced lung metastasis. Real-time PCR, western blot, luciferase assay and ChIP assay were performed to identify the potential targets of Restin and the underlying molecular mechanisms. Among several master regulators of EMT, only ZEB1/2 levels were dramatically inhibited by Restin. Unexpectedly, Restin indirectly regulated ZEB1/2 expression at post-transcriptional level. We further identified mir-200a/b, well-characterized mediators controlling ZEB1/2 expression, were transcriptionally activated by Restin and the regulation was dependent on the p53 binding site in mir-200b/a/429 promoter. Further mechanical studies demonstrated Restin interacted with p73, one of p53 family members, which contributed to Restin-mediated activation of mir-200a/b and suppression of ZEB1/2.

**Conclusions:**

Taken together, our results suggest that Restin inhibits EMT and tumor metastasis by controlling the expression of the tumor metastasis suppressor mir-200a/b via association with p73. Our findings not only establish a mechanistic link between Restin, EMT and tumor metastasis, but also provide strong evidence supporting the notion that MAGE Group II proteins may exert a tumor suppressive effect *in vivo*.

**Electronic supplementary material:**

The online version of this article (doi:10.1186/s12943-015-0370-9) contains supplementary material, which is available to authorized users.

## Background

The melanoma-associated antigen (MAGE) is one of the well-characterized members of the CTA family that contains at least 60 closely related proteins [1], including MAGE-A, B, C, D, E, F, G, H, L2, Necidin, I and J. According to the protein expression patterns and functions, the MAGE family has been divided into groups I and II [2,3]. Group I proteins, including MAGEs A, B and C, are expressed in many types of tumor tissues, but are not expressed in normal tissues [1]. Moreover, their expression is closely correlated with aggressive clinical course, the acquisition of resistance to chemotherapy, the occurrence, and poor clinical outcomes [1,3,4]. Contrast to group I proteins, Group II proteins, such as Necdin and Mage-D1, are universally expressed in all normal tissues but rarely in tumor tissues [5]. They are more likely associated with cell growth inhibition, cell cycle arrest, apoptosis, or cell differentiation [6]. For example, Necdin has been shown to be down-regulated in both carcinoma cell lines and primary tumors [7-9], suggesting Necdin is a potent tumor growth suppressor. Ectopic overexpression of Necdin in a mouse tumor cell line is reported to attenuate tumorigenicity and metastasis *in vivo* [10]. In addition, MAGE-D1 inhibited cell proliferation, migration and invasion of multiple human cancer cells [11]. Although Group II proteins emerge as novel tumor suppressor candidates in a wide range of human cancers, their roles in cancers remain poorly defined.

Restin belongs to MAGE Group II proteins and is known as MAGE H1 [12]. Restin was firstly cloned from the differentiated HL-60 cells induced by all-trans retinoic acid (ATRA) [13], an apoptosis and differentiation inducer. Bioinformatics analysis showed that Restin shared 49% homology with Necdin [14] and both of them were basic proteins. Further analysis found that Restin, Necdin and Mage-D1 had an alkaline conservative region, which is lowly expressed in tumor tissues [14]. Above data indicated that, similar to Necdin and Mage-D1, Restin belongs to Group II proteins. Bioinformatics data from GEO profiles show that Restin is rarely expressed in a variety of cancer cells, while its expression level is pretty high in normal cells. Restin was identified as one of pro-apoptotic genes that determined the response of multiple tumor cells to CD95-mediated apoptosis [15]. Fu HY et al. found that Restin overexpression in Hela cells promoted apoptosis [16]. Denis Selimovic et al. disclosed that Restin overexpression induced apoptosis of melanoma cells via interacting with p75 neurotrophin receptor (p75NTR), leading to the disruption of both NF-ƘB and extracellular signal-regulated kinase (ERK) pathways [12]. Thus, Restin may function as a tumor suppressor, which is similar to Necdin and Mage-D1. Nevertheless, little information is available on its expression patterns and functions, particularly its roles in tumorigenesis *in vitro* and *in vivo*.

The epithelial-mesenchymal transition (EMT) and the reverse process, termed the mesenchymal-epithelial transition (MET), play critical roles in embryogenesis, wound healing, tissue fibrosis, and carcinoma progression [17-20]. EMT is known to be a central mechanism responsible for invasiveness and metastasis of a variety of cancers [19-21]. During tumor development, epithelial cells undergo dynamic cytoskeletal rearrangement, and lose cell adhesion and epithelial components while acquiring mesenchymal and migratory phenotypes [20,22]. Therefore, targeting EMT may serve as an efficient strategy for the treatment of malignant and metastatic tumors.

Our present study, for the first time, demonstrated that Restin remarkably suppressed breast cancer metastasis through inhibiting EMT by controlling the expression and function of the tumor metastasis suppressor mir-200a/b via association with p73 in breast cancer cells.

## Results

### Restin expression level was negatively related with tumor cell mobility

**Figure 1:**
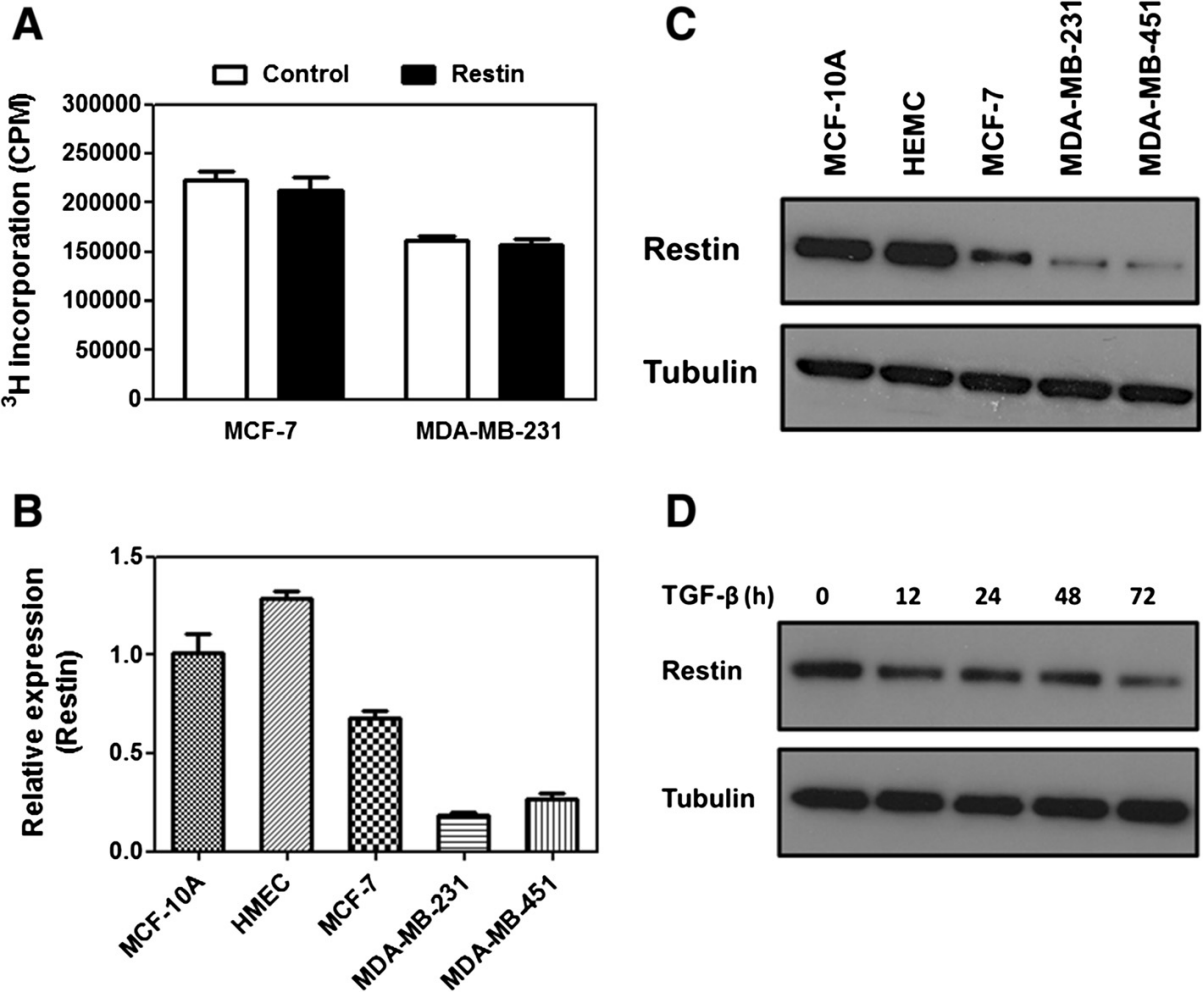
Restin expression level was negatively related with tumor cell mobility. **(A)** Restin overexpression failed to inhibit cell proliferation determined by [^3^H] thymidine incorporation assay. MCF-7 and MDA-MB-231 (2 x 10^4^) cells were seeded onto 24-well plates. [^3^H] thymidine (1 mCi/well) was added 4 hours before the termination of experiment. Data were represented as the means ± S.M. of three independent experiments. mRNA **(B)** and protein **(C)** levels of Restin were determined by RT-PCR and western blot. MCF-10A, HMEC, MCF-7, MDA-MB-231 and MDA-MB-451 cells (1 x 10^5^) were seeded onto 6-well plates. Values were expressed as means ± S.M. of at least three independent experiments. **(D)** Restin expression in MCF-7 cells upon TGF-β treatment was determined by western blot. MCF-7 cells (5 x 10^4^) were seeded onto 6-well plates and TGF-β (5 ng/ml) were added for 6 days. Tubulin was used as a loading control. Results presented here are representative of three different experiments.

Previous study showed that Restin overexpression induced apoptosis of melanoma cells [12], indicating Restin may be associated with cell malignancy. Thus, we first tested if Restin overexpression inhibited cell proliferation in two breast cancer cell lines. Contrast to melanoma cells, Restin overexpression failed to inhibit cell proliferation determined by ^3^[H] incorporation assay (Figure 1A). Next, we detected if Restin was related with cell mobility, another tumor cell malignant feature. To test this, Restin expression level was determined in non-metastatic, low-metastatic and high-metastatic cells. As expected, Restin mRNA level was pretty high in two non-metastatic cells, MCF-10A and HMEC, while it was moderately decreased in low-metastatic MCF-7 cells (Figure 1B). However, Restin was barely detectable in high-metastatic MDA-MB-231 and MDA-MB-451 cells (Figure 1B). Restin protein levels showed the similar trend (Figure 1C). TGF-β is a strong inducer of cell mobility in epithelial cells [23]. As expected, Restin expression level was gradually decreased upon TGF-β treatment in MCF-7 cells (Figure 1D). Above data indicated that Restin expression level is negatively related with tumor cell mobility.

### Restin prevented EMT in breast cancer cells

**Figure 2:**
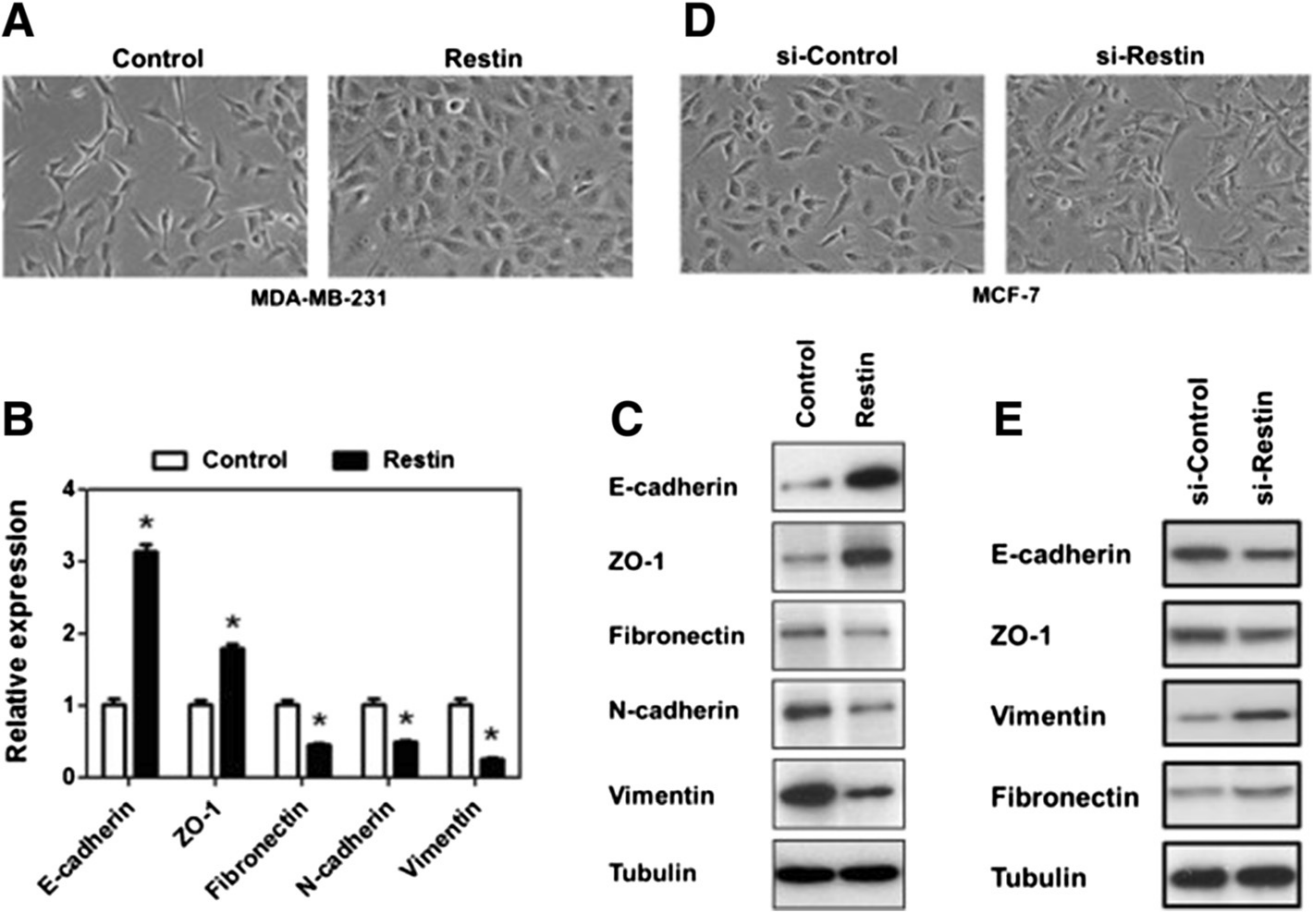
Restin prevented EMT in breast cancer cells. **(A)** Morphologic changes in MDA-MB-231 cells upon Restin overexpression. Phase-contrast cell images were taken (×40 magnification). mRNA **(B)** and protein levels **(C)** of epithelial markers (E-cadherin and ZO-1) and mesenchymal markers (Fibronectin, N-cadherin and Vimentin) in MDA-MB-231 cells (Control and Restin overexpression) were determined by real-time PCR and western blot. Values were expressed as means ± S.M. of at least three independent experiments. * *p* < 0.05 vs Control group. Tubulin was used as a loading control. Results presented here were representative of three different experiments. **(D)** Morphologic changes in MCF-7 cells upon Restin knockdown plus TGF-β treatment. MCF-7 cells (si-Control and si-Restin) were seeded onto 6-well plates and TGF-β (5 ng/ml) were added for 6 days. Phase-contrast cell images were taken (×40 magnification). **(E)** Protein levels of epithelial markers (E-cadherin and ZO-1) and mesenchymal markers (Fibronectin, N-cadherin and Vimentin) in MCF-7 (si-Control and si-Restin) cells were determined by western blot. Tubulin was used as a loading control. Results presented here were representative of three different experiments. Restin: Restin overexpression; si-Restin: Restin knockdown.

EMT is a crucial program for the invasion and metastasis of epithelial tumors that involves loss of cell-cell adhesion and increase of cell mobility [19-21]. To examine if Restin plays a role in EMT process, cell morphology and EMT markers were detected. Restin overexpression and knockdown stable cell lines were generated in MDA-MB-231 and MCF-7 cells and Restin expression levels were confirmed by western blot (Additional file 1: Figure S1). The Control MDA-MB-231 cells retained their elongated spindle-like morphology with sharp borders (Figure 2A). However, Restin overexpressed MDA-MB-231 cells displayed a cobblestone-like epithelial phenotype, grew as a monolayer and formed islands of grouped cells with tight cell-cell contact (Figure 2A). In addition to morphological changes, the expression levels of critical genes for EMT process were determined. E-cadherin plays a central role in maintaining epithelial cell-cell adhesion and polarity [24]. Real-time PCR showed a remarkable increase in E-cadherin expression in Restin overexpressed cells (Figure 2B). The concomitant increase in ZO-1 expression was observed either. In contrast, Restin overexpresssion resulted in a strong repression on the expression of mesenchamal markers, including N-cadherin, Vimentin and Fibronectin, in MDA-MB-231 cells (Figure 2B). Similar results were confirmed by western blot analysis (Figure 2C). Thus, morphological alteration from a fibroblastic to an epithelial phenotype and an increase of E-cadherin level upon Restin overexpression strongly indicate that Restin inhibits EMT process. Furthermore, we determined if knockdown of Restin promoted EMT. To this end, cell morphology and EMT markers were tested upon Restin knockdown in low-mobility MCF-7 cells. Although knockdown of Restin failed to change cell morphology (data not shown), these cells apparently exhibited a spindle-like morphology upon TGF-β induction (Figure 2D). Consistently, E-cadherin and ZO-1 expression were significantly inhibited, whereas Vimentin and Fibronectin expression were moderately enhanced in Restin knockdown cells as compared to si-Control group (Figure 2E). Our data suggest that Restin inhibits EMT in breast cancer cells.

### Restin suppressed migration and invasiveness of breast cancer cells

**Figure 3:**
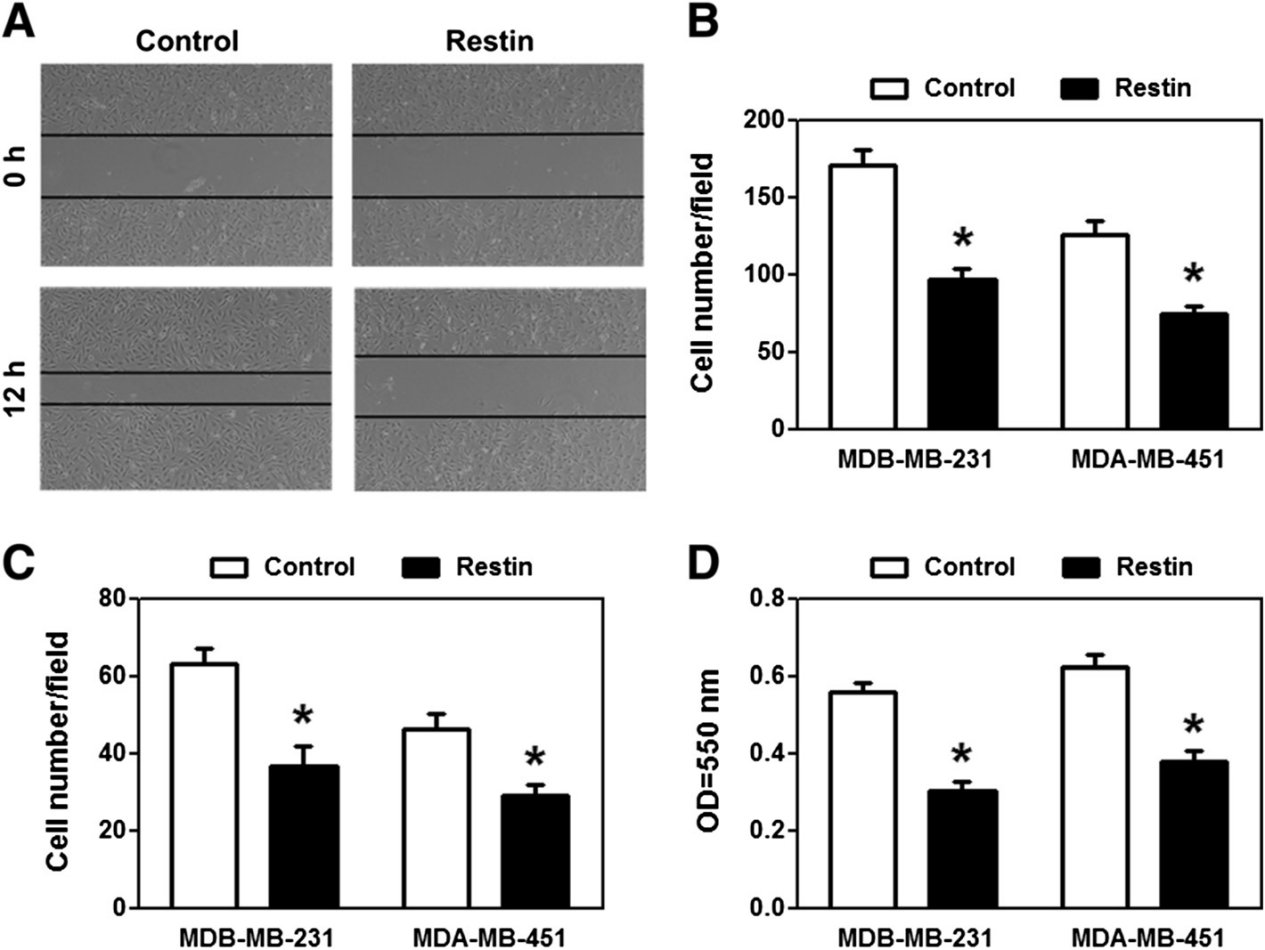
Restin overexpression inhibited migration and invasiveness of breast cancer cells. **(A)** Scratch wound healing assay of MDA-MB-231 cells. 5 × 10^5^ MDA-MB-231 cells (Control and Restin overexpression) were seeded onto 6-well plates. The 6-well plates were scratched (time 0) and 20 random fields of view per well were photographed after 12 h of scratch. Representative pictures from 3 independent experiments were shown. The motility of MDA-MB-231 and MDA-MB-451 cells was evaluated by transwell migration **(B)** and Matrigel invasion assays **(C)** as described in the Materials and Methods. Migration was quantitated by counting 10 fields at a magnification of × 400. Data were presented as the average number of migrated cells from three independent experiments. * *p* < 0.05 vs Control group. **(D)** Adhesion capacity of MDA-MB-231 and MDA-MB-451 cells was measured by cell attachment assay. MDA-MB-231 and MDA-MB-451 cells (Control and Restin overexpression) were seeded onto 96-well plates pretreated with fibronectin for 30 min at a density of 2 × 10^3^ cells/well in triplicate. Cells were allowed to adhere for 2 h and adherent cells were stained with crystal violet and quantified using the absorbance as a measurement at 550 nm. Values were expressed as means ± S.M. of at least three independent experiments. * *p* < 0.05 vs Control.

Fibroblast cells that have undergone MET are characterized by their decreased motility and loss of invasiveness. Therefore, we detected whether Restin overexpression resulted in decreased motility of MDA-MB-231 and MDA-MB-451 cells determined by wound closure assay, transwell migration and invasion assays. As shown in Figure 3A, the percentage of wound closure for Control MDA-MB-231 cells was 50.6%, whereas Restin-overexpressed cells showed 7.43% wound closure, suggesting that Restin severely destroys the migration ability of MDA-MB-231 cells. Consistently, Restin overexpression significantly inhibited the number of migrating cells (43.46% and 40.47% inhibition in MDA-MB-231 and MDA-MB-451 cells, respectively) compared with Control cells (Figure 3B). Next, we performed transwell matrigel invasion assay to assess whether Restin inhibited cell invasiveness. As shown in Figure 3C, Restin overexpression caused a reduction of invasive cells by more than 41.65% and 37.61% in MDA-MB-231 and MDA-MB-451 cells compared with their Control cells. We also found that Restin overexpressed cells displayed significantly less adherence to fibronectin compared with Control group (Figure 3D). These results suggest that Restin suppresses cell migration and invasiveness of metastatic breast cancer cells.

### Restin inhibited lung metastasis *in vivo*

**Figure 4:**
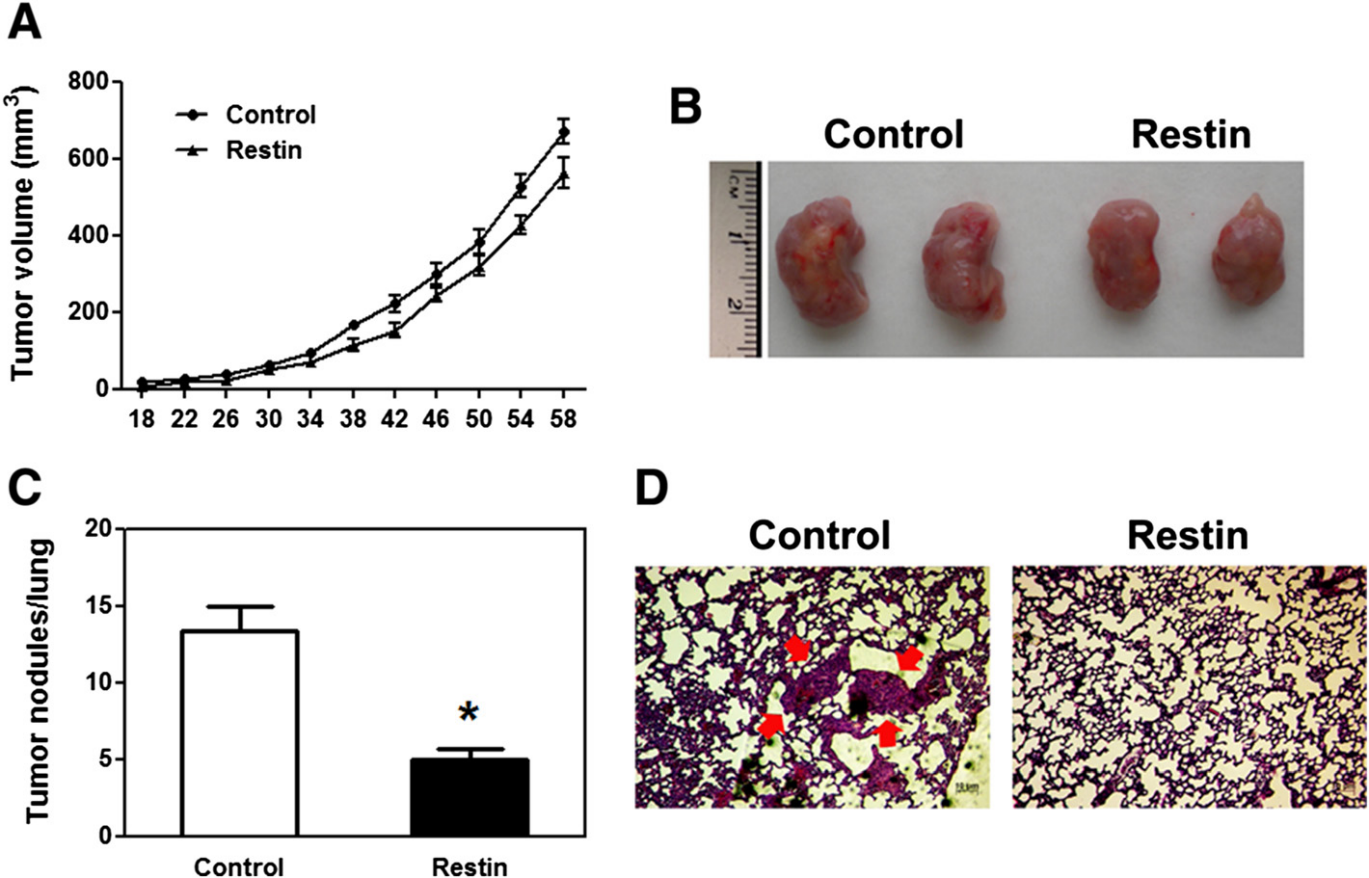
Restin overexpression inhibited lung metastasis *in vivo*. **(A)** The volume of primary tumors was measured after the transplantation of Control and Restin overexpressed MDA-MB-231 cells. 1 × 10^5^ MDA-MB-231 cells were subcutaneously injected into the mammary fat pad of immunocompromised NOD/SCID mice (n = 5). Tumor growth was monitored every 4 days. Data were shown as mean size ± S.M. of tumors in five mice per cell line. Animal experiments were repeated three times. **(B)** Xenograft tumor size from NOD/SCID mice grafted with Control and Restin-overexpressed cells. **(C)** The metastatic nodules in the lungs were counted following the orthotopic transplantation of Control and Restin overexpressed MDA-MB-231 cells. Values were expressed as means ± S.M. in five mice. * *p* < 0.05 vs Control. **(D)** Representative HE staining of lung metastases. Arrows indicate micrometastases.

In order to evaluate if Restin-mediated inhibition of EMT indeed plays a role *in vivo*, tumor growth and lung metastasis were monitored in NOD-SCID mice implanted with Control and Restin overexpressed MDA-MB-231 cells. The tumor growth rate showed no statistic difference between Control and Restin overexpression groups (Figure 4A, *p* > 0.05). Consistently, the tumor size was similar in the two groups (Figure 4B). Interestingly, the tumors formed by Control MDA-MB-231 cells gave rise to 13.32 ± 1.659 metastatic nodules per lung (Figure 4C). However, Restin overexpression resulted in a 2.7-fold decrease of lung metastases (Figures 4C and D). Our *in vivo* data indicate that the morphological changes caused by Restin overexpression is closely related to decreased lung metastasis.

### Restin downregulated ZEB1/2 expression at post-transcriptional level

**Figure 5:**
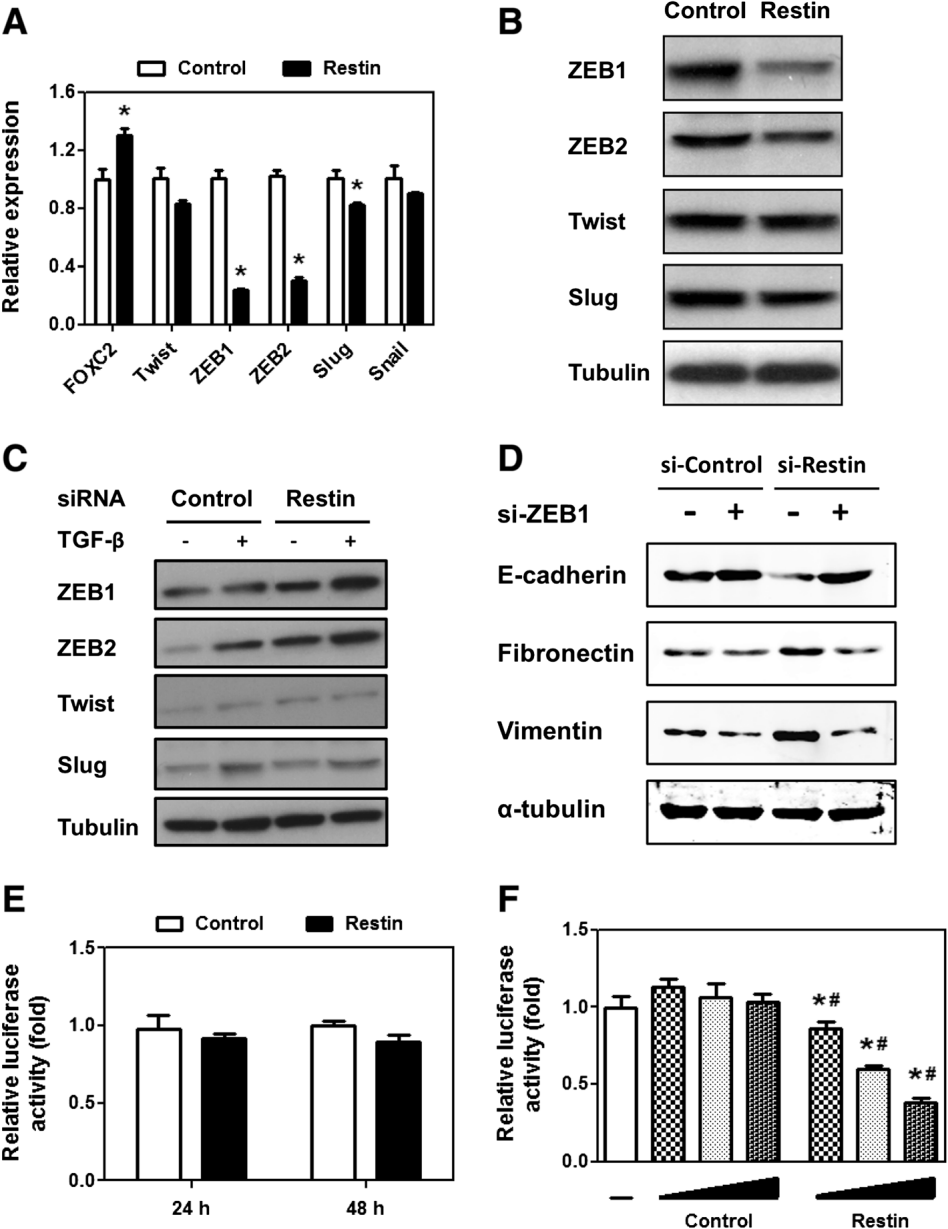
Restin downregulated ZEB1/2 expression at the post-transcriptional level. RNA **(A)** and protein levels **(B)** of FOXC2, Twist, ZEB1, ZEB2, Slug and Snail in Control and Restin overexpressed MDA-MB-231 cells were determined by real-time PCR and western blot. Values were expressed as means ± S.M. of at least three independent experiments. * *p* < 0.05 vs Control group. **(C)** Expression level of above proteins in si-Control and si-Restin MCF-7 cells treated with TGF-β was determined by western blot. Tubulin was used as a loading control. Results presented here were representative of three different experiments. **(D)** MCF-7 stable cells (si-Control and si-Restin) were seed onto 6-well plates and transfected with control and ZEB1 siRNAs (si-ZEB1). 48 h posttransfection, proteins were collected and western blot was performed to detect protein levels of E-cadherin, Fibronectin and Vimentin. Tubulin was used as a loading control. Result presented here was a representative of three different experiments. **(E)** ZEB1 promoter activity was determined by luciferase reporter assay. HEK293 cells were seeded in 24-well plates and transfected with Restin overexpression lentivirus and ZEB1 promoter plasmids. The firefly luciferase activity was normalized to that of the renilla luciferase. **(F)** ZEB1 3’UTR activity was determined in HEK293 cells by luciferase reporter assay upon Restin overexpression. HEK293 cells were seeded in 24-well plates and transfected with ZEB1 3’UTR plasmids and different dose of Restin overexpression lentivirus. The firefly luciferase activity was normalized to that of the renilla luciferase. Values were expressed as means ± S.M. of at least three independent experiments. * *p* < 0.05 relative to cells without addition of lentivirus (−), ^#^ *p* < 0.05 relative to Control group.

Downregulation of E-cadherin is one of the critical markers of EMT in human breast cancers [25]. FOXC2, Twist, ZEB1, ZEB2, Slug and Snail are identified transcription factors that repress E-cadherin transcription and overexpression of each gene induces EMT and promotes malignant cell invasion and dissemination [21,24]. Considering the positive regulation of Restin on E-cadherin, we hypothesize that Restin may control the expression of those transcription factors. We found that the expression levels of ZEB1 and ZEB2 were significantly inhibited by 76.4% and 70.0%, respectively, upon Restin overexpression (Figure 5A). However, a slight reduction in Twist, Snail and Slug mRNA levels was observed (Figure 5A). Consistently, ZEB1 and ZEB2 protein levels were apparently inhibited by Restin overexpression (Figure 5B). However, no apparent difference was observed in Twist and Slug protein levels. On the contrary, Restin knockdown resulted in a dramatic increase in ZEB1 and ZEB2 levels, while only a marginal effect on Twist and Slug expression (Figure 5C, lane 3 versus lane 1). Consistent with TGF-β-mediated negative regulation on Restin, TGF-β treatment further enhanced ZEB1 and ZEB2 expression levels (Figure 5C, lane 4 versus lane 2).

Next, we determined if ZEB1/2 was involved in Restin-mediated inhibition of EMT. E-cadherin, Vimentin and Fibronectin levels were tested upon ZEB1 knockdown (Additional file 1: Figure S2). Control MCF-7 cells transfected with ZEB1 siRNAs showed moderately enhanced E-cadherin and decreased Vimentin and Fibronectin expression (Figure 5D, lane 2 relative to lane 1), which is consistent with ZEB1 function in promoting EMT. Restin knockdown led to reduction of E-cadherin and increase of Vimentin and Fibronectin (Figure 5D, lane 3 relative to lane 1). Additional knockdown of ZEB1 effectively reversed the expression of these proteins, and their levels were comparable to that in Control cells (Figure 5D, lane 4 relative to lane 2). Collectively, these data indicate that ZEB1 is a crucial mediator of Restin-induced phenotypes.

Previous studies showed that Restin was localized in nucleus and it interacted with several transcription factors [26,27], indicating it may regulate ZEB1 expression at the transcriptional level. To test this, ZEB1 promoter activity was detected by luciferase reporter assay. We found that Restin overexpression failed to activate the transcription of ZEB1 (Figure 5E). Since Restin suppressed ZEB1 and ZEB2 mRNA levels, it is possible that Restin may regulate ZEB1 expression at the post-transcriptional level. As expected, Restin overexpression strongly repressed the luciferase activities driven by ZEB1 3’UTR in a dose-dependent manner (Figure 5F). Furthermore, an enhanced luciferase activity was observed upon Restin knockdown (Additional file 1: Figure S3).

### mir-200a/b was responsible for Restin-mediated downregulation of ZEB1

**Figure 6:**
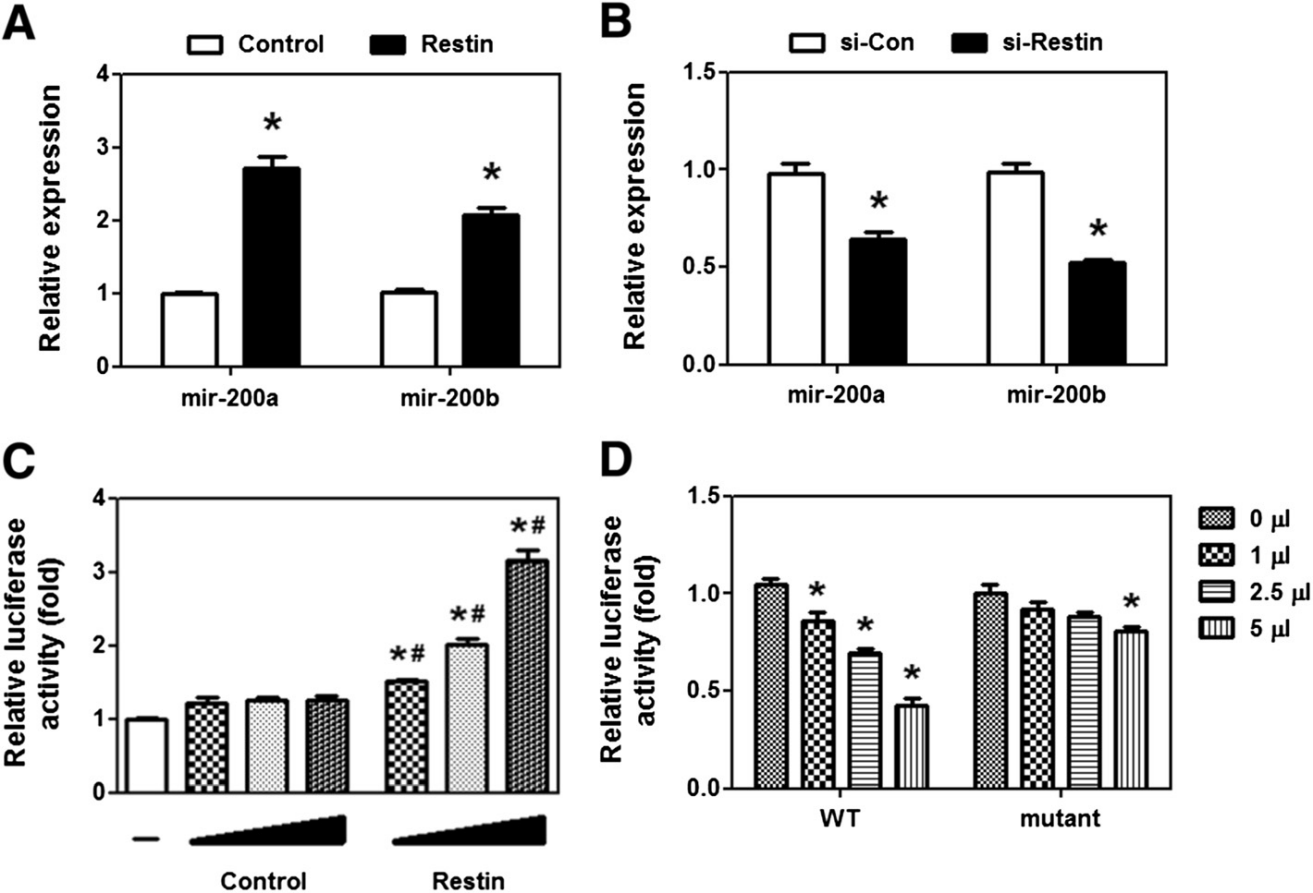
mir-200b/a was responsible for Restin-mediated downregulation of ZEB1. RT-PCR was performed to detect mir-200a and mir-200b levels in Restin overexpressed MDA-MB-231 cells **(A)** and Restin knockdown MCF-7 cells **(B)**. Values were expressed as means ± S.M. of at least three independent experiments. * *p* < 0.05 relative to Control or si-Con group. **(C)** mir-200b/a/429 promoter activity was determined in HEK293 cells by luciferase reporter assay. HEK293 cells were seeded ontn 24-well plates and transfected with mir-200b/a/429 promoter plasmids and different dose of Restin overexpression lentivirus. The firefly luciferase activity was normalized to that of the renilla luciferase. Values were expressed as means ± S.M. of at least three independent experiments. * *p* < 0.05 relative to cells without addition of lentivirus (−), ^#^ *p* < 0.05 relative to their Control group. **(D)** ZEB1 3’UTR (WT and mutant) activity was determined by luciferase reporter assay upon Restin overexpression. WT and mutant ZEB1 3’UTR with deletion of mir-200 binding sites were transfected into HEK293 cells. 24 h later, different dose of Restin overexpression lentivirus were added. The firefly luciferase activity was normalized to that of the renilla luciferase. Values were expressed as means ± S.M. of at least three independent experiments. * *p* < 0.05 relative to cells without addition of Restin lentivirus.

Accumulating evidences demonstrate that mir-200 family is a powerful microRNA family controlling ZEB1/2 expression [23,28]. To determine if mir-200 members are involved in Restin-mediated downregulation of ZEB1/2, mir-200a, mir-200b, mir-429, mir-200c and mir-141 levels were determined upon Restin overexpression and knockdown. As shown in Figure 6A, overexpression of Restin significantly enhanced mir-200a and mir-200b expression by 2.71- and 2.07-fold, while their levels were greatly inhibited by Restin knockdown (Figure 6B), hinting that Restin may regulate the transcription of mir-200b/a/429. However, the expression levels of mir-200c and mir-141, which are located in another genomic cluster, were not altered by Restin overexpression (Additional file 1: Figure S4). Our hypothesis was further confirmed by luciferase reporter assay showing that Restin significantly increased the luciferase activity driven by mir-200b/a/429 promoter but not mir-200c/141 promoter (Figure 6C and Additional file 1: Figure S5). Next we determined if mir-200b/a is indeed responsible for Restin-mediated downregulation of ZEB1 and ZEB2. To this end, a mutant ZEB1 3’UTR with deletion of mir-200b binding sites was constructed. Consistent with above data, Restin overexpression decreased the luciferase activities driven by ZEB1 3’UTR, while failed to reduce the luciferase activities driven by mutant ZEB1 3’UTR (Figure 6D).

### Restin activated mir-200b/a/429 transcription in a p73-dependent manner

**Figure 7:**
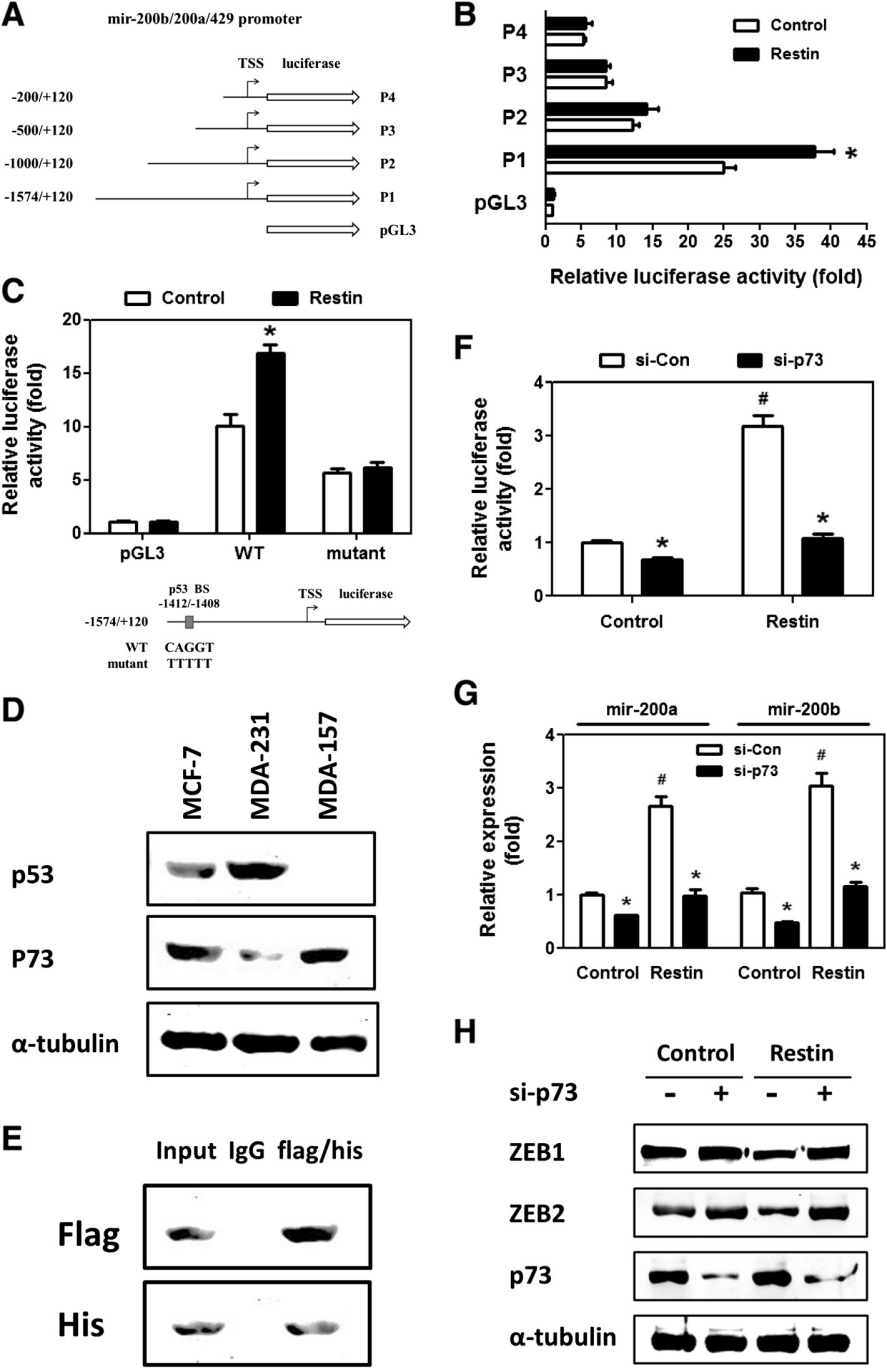
**Restin activated mir-200 transcription in a p73-dependent manner. (A)** A schematic diagram of the human mir-200b/a/429-luciferase constructs (WT and its truncations) was shown. **(B)** A series of mir-200b/a/429 promoter activities were determined by luciferase reporter assay. Restin overexpression lentivirus were added into 293T cells and mir-200b/a/429 promoter truncation plasmids were transiently transfected. * *p* < 0.05 relative to Control lentivirus. **(C)** mir-200b/a/429 promoter (WT and p53 mutant) activities were measured by luciferase reporter assay. * *p* < 0.05 relative to Control lentivirus. **(D)** Western blot was performed to detect p53 and p73 levels in MCF-7, MDA-MB-231 and MDA-MB-157 cells. **(E)** Co-immunoprecipitation assay was performed to detect the exogenous interaction between Restin and p73. HERK-293 cells were transiently transfected with Flag-tagged Restin and His-tagged p73 plasmids. (Upper panel) Cell extracts were immunoprecipitated with mouse IgG or anti-His antibody and then blotted with anti-Flag antibodies. (Lower panel) Cell extracts were immunoprecipitated with mouse IgG or anti-Flag antibody and then blotted with anti-His antibody. **(F)** MDA-MB-231 stable cells (Control and Restin overexpression) were seed onto 24-well plates and co-transfected with Control or p73 siRNAs (si-Con and si-p73) and mir-200b/a/429 promoter construct. * *p* < 0.05 relative to si-Con group, # *p* < 0.05 relative to Control cells. **(G)** MDA-MB-231 stable cells (Control and Restin overexpression) were seed onto 6-well plates and transfected with control and p73 siRNAs (si-Con and si-p73). RT-PCR was performed to detect mir-200a and mir-200b expression levels. * *p* < 0.05 relative to si-Con group, # *p* < 0.05 relative to Control cells. **(H)** MDA-MB-231 stable cells (Control and Restin overexpression) were seed onto 6-well plates and transfected with control and p73 siRNAs (si-p73). Western blot was performed to detect ZEB1 and ZEB2 levels.

To investigate the molecular mechanisms by which Restin upregulated mir-200b/a transcription, a series of mir-200b/a/429 promoter truncations were constructed (Figure 7A) and their luciferase activities were determined. As shown in Figure 7B, Restin dramatically enhanced the luciferase activities driven by WT mir-200b/a/429 promoter (P1,-1574/+120). However, Restin failed to activate the mir-200b/a/429 promoter activities with deletion of p53 binding sites (-1412/-1408) in P2 truncation (-1000/+120) and another two shorter truncations (P3 and P4). Above data suggest that p53 binding site is critical for Restin-mediated activation of mir-200b/a/429 transcription. To further demonstrate this, we introduced a luciferase reporter construct in which the p53 binding site had been mutated (Figure 7C, bottom panel). As expected, the luciferase activities driven by WT mir-200b/a/429 promoter was increased by 1.69-fold upon Restin overexpression, whereas Restin was unable to upregulate the activities of mir-200b/a/429 promoter with mutations in p53 binding site (Figure 7C). These data indicate that Restin directly or indirectly activates the transcription of mir-200b/a/429 through p53 binding site on its promoter.

The p53 family comprises three genes that encode for p53, p63 and p73 [29]. To identify which factor is involved in Restin-activated mir-200b/a/429 transcription, the expression levels of three proteins were first detected in several breast cancer cell lines. It is well-documented that MCF-7 cells contain wild-type p53, MDA-MB-231 cells carry mutant p53 with greater levels, and MDA-MB-157 cells expressed no p53 [30]. As shown in Figure 7D, p53 was moderately expressed in MCF-7 cells, and was undetectable in MDA-MB-157 cells, whereas a high level of mutant p53 protein was observed in MDA-MB-231 cells. All cell lines were negative for p63 (data not shown), whereas contained detectable and comparable p73 levels (Figure 7D). It has been demonstrated that MDA-MB-231 cells have mutant p53 due to an arginine to lysine mutation at position 280 and the mutant p53 does not retain the tumor suppressive ability of wild-type p53 [30]. We compared the luciferase activities driven by mir-200b/a/429 promoter in above three cell lines and found that Restin activated the luciferase activities in a comparable manner whatever the expression level and function of p53 in those cells (Additional file 1: Figure S6). Therefore, we postulate that p53 may not participate in Restin-mediated transcriptional activation of mir-200b/a/429. Considering the undetectable level of p63, we hypothesize that p73 may play a role in Restin-mediated upregulation of mir-200b/a. To test this, reciprocal co-immunoprecipitation was performed to detect the interaction between p73 and Restin. HERK-293 cells were transiently transfected with Flag-tagged Restin and His-tagged p73 plasmids. Upon immunoprecipitation of p73 using an anti-His antibody, Restin was coimmunoprecipitated (Figure 7E, upper panel). Likewise, in the immunoprecipitate of Flag-Restin, p73 protein was recovered (Figure 7E, lower panel). Endogenous interaction of p73 and Restin was also examined in MCF-7 cells. Anti-Restin antibody could co-immunoprecipitate endogenous p73 (Additional file 1: Figure S7). As expected, endogenous interaction between p53 and Restin was not found in MCF-7 cells determined by co-immunoprecipitation assay (Additional file 1: Figure S8).

Next we investigated if Restin upregulated mir-200b/a transcription in a p73-dependent manner. siRNAs mediated p73 knockdown was confirmed by western blot (Additional file 1: Figure S9). In MDA-MB-231 Control cells, knockdown of p73 inhibited the luciferase activities driven by mir-200b/a/429 promoter by 0.69-fold, suggesting that p73 indeed regulates the transcription of mir-200b/a/429 (Figure 7F, lane 2 relative to lane 1). In Restin overexpressed cells, the luciferase activity was dramatically increased by 3.20-fold compared to Control cells (Figure 7F, lane 3 relative to lane 1). However, its activity was significantly decreased by 2.93-fold upon p73 knockdown (Figure 7F, lane 4 relative to lane 3) and reached to the level exhibited by Control cells plus p73 knockdown (Figure 7F, lane 4 relative to lane 2). Consistently, we found that p73 knockdown slightly decreased mir-200a/b expression level which was apparently enhanced by Restin overexpression (Figure 7G). However, p73 knockdown remarkably diminished mir-200a/b expression to the similar level as seen in the MDA-MB-231 Control cells (Figure 7G). To further confirm the contribution of p73 to Restin-mediated regulation of mir-200b/a, levels of ZEB1/2, targets of mir-200b/a, were evaluated upon p73 knockdown. Although Restin overexpression apparently inhibited ZEB1/2 expression (Figure 7H, lane 3 relative to lane 1), p73 knockdown almost restored ZEB1/2 expression to the levels observed in Control cells (Figure 7H, lane 4 relative to lane 2), suggesting that p73 is a critical mediator for Restin-induced upregualtion of mir-200b/a and downregulation of ZEB1/2.

## Discussion

MAGE group II proteins function as putative tumor suppressors in a variety of cancers [5,6]. However, its clinical effects on tumor development and the underlying mechanisms are poorly characterized. Restin is a novel member of MAGE group II proteins [12]. Selimovic et al. demonstrated Restin induced apoptosis of melanoma cells via interaction with p75 neurotrophin receptor [12]. Wang et al. showed that p53 upregulated Restin expression at the transcriptional level [31]. All current data hint that Restin may be involved in tumor cell growth. Nevertheless, the molecular mechanisms underlying the repression of Restin in tumor cells and its role in tumor development had not been reported yet. Here, we, for the first time, showed that Restin suppressed EMT and lung metastasis by activating the transcription of mir-200b/a via association with p73. Considering the pivotal roles of mir-200b/a and p73 in tumorigenesis, our studies provide significant insights that Restin may participate in other functions closely related to mir-200b/a and p73, such as EMT, cancer stem cells, tumor angiogenesis and drug resistance.

mir-200 family plays critical roles in tumor development and progression through inhibiting EMT and suppressing tumor invasion by directly repressing the transcription factors ZEB1 and ZEB2 [23,28,32,33]. Previous works on mir-200 regulation have largely focused on mechanisms of transcriptional inhibition, epigenetic modification and transcriptional activation of this miRNA family, and identified multiple factors regulating mir-200 expression, including ZEB1, ZEB2, HDAC, p53 family [32]. Our study demonstrated that Restin upregulated mir-200 expression at the transcriptional level, indicating Restin may serve as a novel regulator of mir-200. However, Restin-mediated upregulation of mir-200b/a/429 was dependent on p53 binding site in its promoter, indicating p53 family members (p53, p63, p73) are involved in above process. p53 suppresses EMT by transactivating the expression of mir-200 family members in primary hepatocellular carcinomas (HCCs) and 9 HCC cell lines [34]. Simlar to p53, p73 and p63 can directly associate with the mir-200b/a/429 promoter and activate mir-200 transcription in ovarian carcinoma cells [35]. Although MDA-MB-231 cells or MDA-MB-157 cells possess mutant p53 or no p53 expression, Restin still activated mir-200b/a/429 transcription, indicating p53 protein may not participate in above process and Restin may not associate with p53. Our hypothesis was proven by our data showing that no endogenous interaction between Restin and p53 was found. By performing Co-IP experiment, we found that Restin can closely interact with p73. Moreover, p73 knockdown diminished Restin-mediated regulation on mir-200a/b and ZEB1/2. Thus, our observations provide new insights into the role of p53 family members in mediating Restin’s function in tumorigenicity.

Based on the chromosomal locations, the mir-200 family can be divided into two clusters: the mir-200b/a/429 cluster containing mir-200a, mir-200b and mir-429, and the mir-200c/141 cluster, which contains mir-200c and mir-141 [32]. Although both clusters contained putative p53 binding sites, Restin drove mir-200b/a/429 transcription rather than that of mir-200c/141 via association with p73. Interestingly, a recent report found that in mammary epithelial cells, p53 serves as a transcriptional activator of mir-200c/141, but not of the mir-200b/a/429 [36]. We speculate that the transcription of mir-200 clusters by p53 family members may be dependent on the expression level and functions of p53 family members, the promoter methylation status of mir-200 promoters or other transcriptional co-factors, which need to be further elucidated.

p53 is involved in tumor initiation as well as tumor progression [37-39]. However, deletions or mutations of p53 are frequently found in cancers [39-43]. p63 and p73 are rarely mutated in a large number of tumors examined to date [42]. Therefore, in the absence of p53, these proteins may be involved in the control of the ZEB/mir-200 equilibrium and EMT-MET plasticity. Our data showed that Restin interacted with p73 rather than p53 and upregulated mir-200b/a expression even in p53 mutant cells. Our results shed light on the transcriptional regulation of Restin on mir-200s, which has potential therapeutic value in the development of approaches aimed at modifying mir-200s expression for the treatment of diverse forms of human cancers, especially those cancers with p53 mutation.

Restin was identified from HL-60 cells induced by all-trans-retinoic acid (ATRA). Restin expression level was undetectable in HL-60 cells, whereas its level was sharply increased upon ATRA treatment [13,14]. Consistently, we observed that Restin expression was pretty high in normal breast epithelial cells compared to breast cancer cells. Its expression in low-metastatic cells was higher than that in high-metastatic cells, hinting that Restin expression level is negatively related to tumor cell malignancy. Our data was supported by current evidence showing that Necdin, another member of MAGE group II proteins, was expressed at a low level in MDA-MB-468 and MDA-MB-231 cells, but exhibited much higher levle in low-metastatic MCF-7 cells [8]. Contrast to Restin and Necdin, MAGE-A and C, belong to MAGE group I proteins, exerted an oncogenic role in breast cancer and exhibited a positive correlation with tumor malignancy [1,3,4]. Considering the undetectable expression of Restin in high-metastatic MDA-MB-231 cells and the dramatic inhibition of tumor metastasis by Restin overexpression, we hypothesize that Restin may function as a tumor-suppressor in breast cancers. Our ongoing studies are being performed to detect Restin expression in normal breast tissues, para-carcinoma tissues and carcinoma tissues and to statistically analyze the association of its expression level with tumor malignant grade, tumor metastasis, relapse, and clinical prognosis, which will strongly support our hypothesis.

## Conclusions

Taken together, our study, for the first time, demonstrated that Restin strongly inhibited lung metastasis *in vivo*, which strongly suggests that Restin may serve as a potent tumor suppressor in breast cancers although our study is still in a preliminary stage. Our current data not only shed a light on the function of Restin in tumor development in light of its inhibition on EMT process but also facilitate other researchers to extend or verify its function in other type of cancers.

## Materials and methods

### Cell culture and reagents

MCF-7, MDA-MB-157, MDA-MB-231 and MDA-MB-451 breast cancer cells were grown in Dulbecco’s modified Eagle’s medium (Gibco, Grand Island, NY) supplemented with 10% fetal bovine serum, 2 mM glutamine, 100 U/ml penicillin, and 100 μg/ml streptomycin at 37°C in a humidified atmosphere of 5% CO2. MCF-10A, a spontaneously immortalized human breast epithelial cell line, was maintained in DMEM/F12 medium (Invitrogen Life Technologies, Carlsbad, CA). Human mammary epithelial cells (HMEC) were cultured in mammary epithelial cell complete medium (Invitrogen). All cell lines were obtained from the ATCC. p73 and ZEB1 siRNAs (Stealth™ siRNAs) were synthesized from Invitrogen and were dissolved in DEPC-treated H_2_O at a concentration of 20 pmol/μl as a stock.

### Generation of Restin overexpression and knockdown stable cell lines

Restin coding region was amplified by PCR using MCF-7 cDNA as a template and inserted into PmeI restriction enzyme sites of pWPI-GFP lentivirus expression vector by using the following primers: 5’-gtacgtttaaac*atgcctcgggagcgaaagag*-3’ and 5’-gatcgtttaaac*ttaaggggcggaataacccc*-3’. siRNA duplexes targeting Restin were annealed and inserted into Mlu1 and Cla1 restriction enzyme sites of pLVTHM-GFP lentivirus expression vector. Forward: 5’-cgcgtcccc *ggaatcgagcaaactgaaa*ttcaagaga*tttcagtttgctcgattcc*tttttggaaat-3’; Reverse: 5’-cgatttccaaaaa*ggaatcgagcaaactgaaa*tctcttgaa*tttcagtttgctcgattcc*gggga-3’. The PCR products were confirmed by sequencing. Lentivirus were produced according to the manufacture’s instruction. MCF-7 and MDA-MB-231 cells were seeded onn 6-well plates and transduced with 10 μl lentivirus. 24 h later, transfected cells were trypsinized and reseeded into 10-cm culture plates. We refer to these cells in the text as Control and Restin (overexpression), si-Control and si-Restin (knockdown).

### Constructs

The following plasmids were bought from Addgene (Cambridge, MA). mir-200b-200a-429 promoter (Plasmid 35539: pGL3-1574/+120), mir-200c-141 (Plasmid 35534), ZEB1 3’UTR (Plasmid 35535: pCI-neo-RL-ZEB1), ZEB1 3’UTR with mutation in mir-200b seeding region (Plasmid 35537: pCI-neo-RL-ZEB1 200bmutx5). ZEB1 promoter plasmid (HPRM23421) was bought from GeneCopoeia (Rockville, MD). 2000 bp of mir-200c–141 promoter region was amplified and inserted into pGL3 vector using Nhe I and Bgl II sites. A series of mir-200 promoter truncations were amplified by using mir-200 promoter plasmid (pGL3-1574/+120) as a template and cloned into the pGL3-basic (Promega) reporter using Nhe I and Bgl II sites. The primer sequences were as follows: pGL3-1000/+120: forward, 5’-gt*gctagc*GAAAACCGTGGGGTCCGCTG-3’; pGL3-200/+120: forward, 5’-ac*gctagc*AAGGTGGGGGCGGGACGGAG-3’; reverse, 5’-ct*agatct*CCTGGCACAGGAAGTCAGTTC-3’. Mutation in the p53 binding site was made by using the Quikchange Multi site–directed mutagenesis kit (Stratagene, La Jolla, CA) using the primer pairs 5’-CCAGCTCCCAGG***TTTTT***CCCGCCG -3’ and 5’-CGGCGGG***AAAAA***CCTGGGAGCTGG-3’ (p53 mutant) (the mutation sites were shown in bold) . Restin and p73 coding regions were amplified using MCF-7 cDNA as a template and inserted into our previously recombined pcDNA3.1 (+)-flag and pcDNA3.1 (+)-his vectors, respectively.

### *In vivo* animal experiments

NOD/SCID mice were purchased from Beijing HFK Bioscience CO., LTD (Bejing, China). All animal experiments were performed under the approval of Institutional Animal Care and Use Committee (IACUC) at Henan Cancer Hospital (Permit No: 2014ct001). 1 × 10^6^ MDA-MB-231 cells were resuspended in 20 μl PBS and subcutaneously injected into the fourth mammary fat pad of 8-week old female NOD/SCID mice (n=5 mice/group). Primary tumor growth was evaluated every four days by caliper, and tumor volume was estimated using the following formula: (*L* × *W*^2^)/2. Metastatic growth in the lung was allowed to develop for 6–8 weeks. Primary tumors and lung tissues were fixed in 10% neutral buffered formalin, embedded in paraffin, and subjected to standard hematoxylin and eosin (H & E) staining.

### Transfection and luciferase reporter assay

Cells were seeded onto 24-well plates and transfected with 800 ng of luciferase vectors (ZEB promoter, mir-200 promoter, ZEB 3’UTR (WT and mutant)) per well using Lipofectamine^2000^ Transfection Reagent (Invitrogen). 50 ng of pBind plasmids were cotransfected to normalize for transfection efficiency. 24 h later, cells were lysed with PLB lysing buffer. The luciferase activities were measured with the dual-luciferase reagent assay kit (Promega, Madison, WI) according to the manufacturer’s instructions using the TD-20/20 Luminometer (Turner Designs). All reporter assays are shown as relative luciferase activities (averaged ratios of Firefly luciferase: Renilla FSE) and are representative of three experiments.

### Quantitative real-time PCR

RNAs were collected using Trizol Reagent (Invitrogen). Reverse transcription was carried out using 2 μg RNA in a 20 μl reaction volume using the Superscript III Reverse Transcription Kit (Invitrogen). Real-time PCR was performed with the SYBR® Premix Ex Taq^TM^ (Takara Biotechnology, Dalian, China) in 20 μl reactions using ABI PRISM ® 7500 Real-Time PCR System (Applied Biosystems, Grand Island, NY). GAPDH was used as an internal control. Primer sequences are listed in the Additional file 1: Table S1. For mir-200 detection, RNAs were reverse transcribed by miScript Reverse Transcription Kit (Qiagen, Valencia, CA). qPCR of mir-200a and 200b was performed using miScript SYBR Green PCR Kits (Qiagen). mir-200 expression was normalized with U6.

### Western blot

MDA-MB-231 cells were harvested and lysed in a RIPA buffer containing 50 mM Tris–HCl, pH 7.5, 150 mM NaCl, 1% NP-40, 0.1% SDS, 0.5% sodium deoxycholate, and 50 mM NaF. One tablet of protease inhibitor mixture (Complete Mini, Roche Applied Science) was added just prior to use. Then 30 μg protein lysates were separated by 12% SDS–PAGE and transferred onto nitrocellulose. After blocking in a 5% non-fat dried milk solution in washing buffer containing 10 mmol/l Tris (pH 7.5), 50 mmol/l NaCl, and 0.02% Tween 20 (TBST), membranes were incubated overnight at 4°C with primary antibodies. After washed three times with TBST, membranes were incubated for 1 h with horseradish peroxidase-coupled secondary antibodies at room temperature. Signals were detected with the ECL kit (Pierce, Rockford, IL).

### Antibodies

The following antibodies were used: rabbit polyclonal anti-Slug (1:1000), anti-Twist1 (1:500), anti-ZEB1 and anti-ZEB2 (1:500, Santa Cruz Biotechnology, Santa Cruz, CA). Rabbit monoclonal anti-E-cadherin (1:1000, Santa Cruz). Rabbit polyclonal anti-MDM2 (C-18) (1:500, Santa Cruz). Mouse monoclonal anti-α-tubulin (1:2000) and anti-fibronectin (1:1000) (Cell Signaling, Danvers, MA). Mouse monoclonal anti-N-cadherin (1:500), anti-ZO-1 (1:1000) and anti-vimentin (1:1000) (Abcam, Cambridge, UK). Mouse monoclonal anti-Restin (1:500), anti-His (1:1500) and anti-Flag (1:2000) (Sigma). Mouse monoclonal anti-p73 (1:1000), anti-p53 (1:1000) and anti-p63 (1:1000) (Abcam). Horseradish peroxidase–conjugated secondary antibodies were obtained from Amersham Biosciences.

### Co-immunoprecipitation assay

Human 293 T cells were seeded onto the 10-cm plates and cotransfected with Flag-tagged Restin and His-tagged p73 constructs using Lipofectamine^2000^ Transfection Reagent (Invitrogen). 2 days after transfection, cells were lysed in precooled RIPA buffer for 30 min on ice, followed by 15 min of centrifugation. Cell lysates were precleaned with protein G-Sepharose beads (GE Healthcare) for 30 min at 4°C. After centrifugation at 14,000 rpm at 4°C for 15 min, cleaned lysates were incubated with either anti-Flag or anti-His antibody on a rocking platform for 4 h at 4°C. Protein complexes were immunoprecipitated by incubation with protein G-Sepharose beads for 30 min at 4°C and then washed three times with lysis buffer. Immunoprecipitated proteins were eluted with 50 μl of Laemmli loading buffer and separated by 10% SDS-PAGE. Protein expression was evaluated by Western blot analysis with either His or Flag monoclonal antibodies.

### Wound closure assay

MDA-MB-231 cells were seeded onto 6-cm cell culture dishes at a density of 5 × 10^5^. A wound was incised in the central area of the confluent culture by a 10 μl pipette tip. 12 h after scratch, phase-contrast pictures were taken by a Nikon microscope using a 10x phase contrast objective.

### Cell migration and invasion assays

2 × 10^4^ MDA-MB-231 or MDA-MB-451 cells resuspended in 200 μl serum-free medium were seeded in the upper chamber with serum-containing medium in the lower chamber of 24-well transwell plates (BD Biosciences, San Jose, CA). After 6 h, the experiment was terminated by wiping the cells from the wells with a cotton swab and fixed and stained with 0.05% crystal violet for 2 h. Matrigel invasion assays were done using the BD BioCoat Matrigel Invasion Chamber (BD Biosciences). The procedures and the analyses were the same as those for the transwell migration assay except for the presence of the Matrigel.

### Cell adhesion assay

96-well cell culture plates were pretreated with 20 μg/ml fibronectin at 37°C for 2 h and blocked with 0.5% BSA solution for 1 h. MDA-MB-231 or MDA-MB-451 cells were plated onto 96-well cell culture plates at a density of 1×10^4^ cells/well. Cells were allowed to adhere for 30 min, after which the media were removed and nonadhered cells were rinsed away. Adhered cells were fixed with 4% paraformaldehyde, stained with crystal violet for 10 min and dissolved in 2% SDS. The absorbance was measured at 550 nm with an ELISA reader (Bio-Tek, Winooski, VT).

### [^3^H] thymidine incorporation assay

MCF-7 and MDA-MB-231 cells (5 × 10^4^) (control and Restin overexpressed cells) were seeded onto the 24-well plates. [^3^H] thymidine (1 mCi/well) was added 4 hours before the cells were harvested and thymidine incorporation was measured by scintillation counting (PerkinElmer, Waltham, MA).

### Statistical analyses

Statistical significance of the studies was analyzed by Student’s *t* test. Differences with *P* values of <0.05 are considered significant.

## Additional file

Additional file 1: Table S1. Primers used for quantitative real-time PCR. **Figure S1.** Western blot was performed to confirm Restin expression levels in Restin overexpressed MDA-MB-231 cells and Restin knockdown MCF-7 cells. **Figure S2.** Western blot was performed to detect ZEB1 expression levels in cells transfected with negative control and ZEB1 siRNAs. **Figure S3.** ZEB1 3’UTR activity was determined in HEK293 cells by luciferase reporter assay upon Restin knockdown. HEK293 cells were seeded onto 24-well plates and transfected with ZEB1 3’UTR plasmids and different dose of Restin knockdown lentivirus (si-Restin). **Figure S4.** mir-200c and mir-141 levels were determined in Control and Restin overexpressed MDA-MB-231 cells by real-time PCR. **Figure S5.** mir-200c/141 promoter activity was determined by luciferase reporter assay. **Figure S6.** mir-200b/a/429 promoter activities were measured by luciferase reporter assay in multiple cell lines. **Figure S7.** Co-immunoprecipitation assay was performed to detect the endogenous interaction between Restin and p73. (Upper panel) MCF-7 cell extracts were immunoprecipitated with mouse IgG or anti-p73 antibody and then blotted with anti-Restin antibody. (Lower panel) Cell extracts were immunoprecipitated with mouse IgG or anti-Restin antibody and then blotted with anti-p73 antibody. Input, total cell lysates. **Figure S8.** Co-immunoprecipitation assay was performed to detect the endogenous interaction between Restin and p53. MCF-7 cell extracts were immunoprecipitated with mouse IgG or anti-p53 antibody and then blotted with anti-Restin (upper panel) and anti-MDM2 (C-18) antibodies (lower panel). MDM2 p90 is a positive control. Input, total cell lysates. **Figure S9.** Western blot was performed to detect p73 expression levels in cells transfected with negative control and p73 siRNAs.

## Abbreviations

EMT: Epithelial-mesenchymal transition
ATRA: All-trans retinoic acid
MAGE: Melanoma associated antigen
HMEC: Human mammary epithelial cells
H & E: Hematoxylin and eosin
RT-PCR: Real-time PCR
