# Supplementary material for: Restin suppressed epithelial-mesenchymal transition and tumor metastasis in breast cancer cells through upregulating mir-200a/b expression via association with p73
Source: Mol Cancer. 2015 May 14;14:102. doi: 10.1186/s12943-015-0370-9 (PMC4429374; doi:10.1186/s12943-015-0370-9)
Supplement: Additional file 1: Table S1. — Primers used for quantitative real-time PCR. Figure S1. Western blot was performed to confirm Restin expression levels in Restin overexpressed MDA-MB-231 cells and Restin knockdown MCF-7 cells. Figure S2. Western blot was performed to detect ZEB1 expression levels in cells transfected with negative control and ZEB1 siRNAs. Figure S3. ZEB1 3’UTR activity was determined in HEK293 cells by luciferase reporter assay upon Restin knockdown. HEK293 cells were seeded onto 24-well plates and transfected with ZEB1 3’UTR plasmids and different dose of Restin knockdown lentivirus (si-Restin). Figure S4. mir-200c and mir-141 levels were determined in Control and Restin overexpressed MDA-MB-231 cells by real-time PCR. Figure S5. mir-200c/141 promoter activity was determined by luciferase reporter assay. Figure S6. mir-200b/a/429 promoter activities were measured by luciferase reporter assay in multiple cell lines. Figure S7. Co-immunoprecipitation assay was performed to detect the endogenous interaction between Restin and p73. (Upper panel) MCF-7 cell extracts were immunoprecipitated with mouse IgG or anti-p73 antibody and then blotted with anti-Restin antibody. (Lower panel) Cell extracts were immunoprecipitated with mouse IgG or anti-Restin antibody and then blotted with anti-p73 antibody. Input, total cell lysates. Figure S8. Co-immunoprecipitation assay was performed to detect the endogenous interaction between Restin and p53. MCF-7 cell extracts were immunoprecipitated with mouse IgG or anti-p53 antibody and then blotted with anti-Restin (upper panel) and anti-MDM2 (C-18) antibodies (lower panel). MDM2 p90 is a positive control. Input, total cell lysates. Figure S9. Western blot was performed to detect p73 expression levels in cells transfected with negative control and p73 siRNAs. [file 12943_2015_370_MOESM1_ESM.doc]

| Restin | 5’-gcaaaatccaggcctcagag-3’ | 5’-cccatgatgaagatgagcgc-3’ |
| --- | --- | --- |
| E-cadherin | 5’-ggttattcctcccatc agct-3’ | 5’-cttggctgaggatggtgta-3’ |
| ZO-1 | 5’-ttttcctgcttgacctccct-3’ | 5’-aacacggaacacctctcctt-3’ |
| Fibronectin | 5’-ggacatgcattgcctactcg-3’ | 5’-gaatcctggcattggtcgac-3’ |
| Vimentin | 5’-gagtccactgagtaccggag-3’ | 5’-acgagccatttcctccttca-3’ |
| N-cadherin | 5’-cggtttcatttgagggcaca-3’ | 5’-ttggagcctgagacacgatt-3’ |
| Snail | 5’-ttaccttccagcagccctac-3’ | 5’-tcccactgtcctcatctgac-3’ |
| Slug | 5’-tgcctgtcataccacaacca-3’ | 5’-gaggtgtcagatggaggagg-3’ |
| FOXC2 | 5’-aaggtggtgatcaagagcga-3’ | 5’-ggtcatgatgttctccacgc-3’ |
| Twist | 5’-agtcttacgaggagctgcag-3’ | 5’-aggaagtcgatgtacctggc-3’ |
| ZEB1 | 5’-tcccacacgaccacagatac-3’ | 5’-ctgaggagaactggttgcct-3’ |
| ZEB2 | 5’-atggcctacacctacccaac-3’ | 5’-tttgcgagacagacaggagt-3’ |
| β-actin | 5’-gctcgtcgtcgacaacggctc-3’ | 5’-caaacatgatctgggtcatcttctc-3’ |

**Table 1. Primers used for quantitative real-time PCR**

**
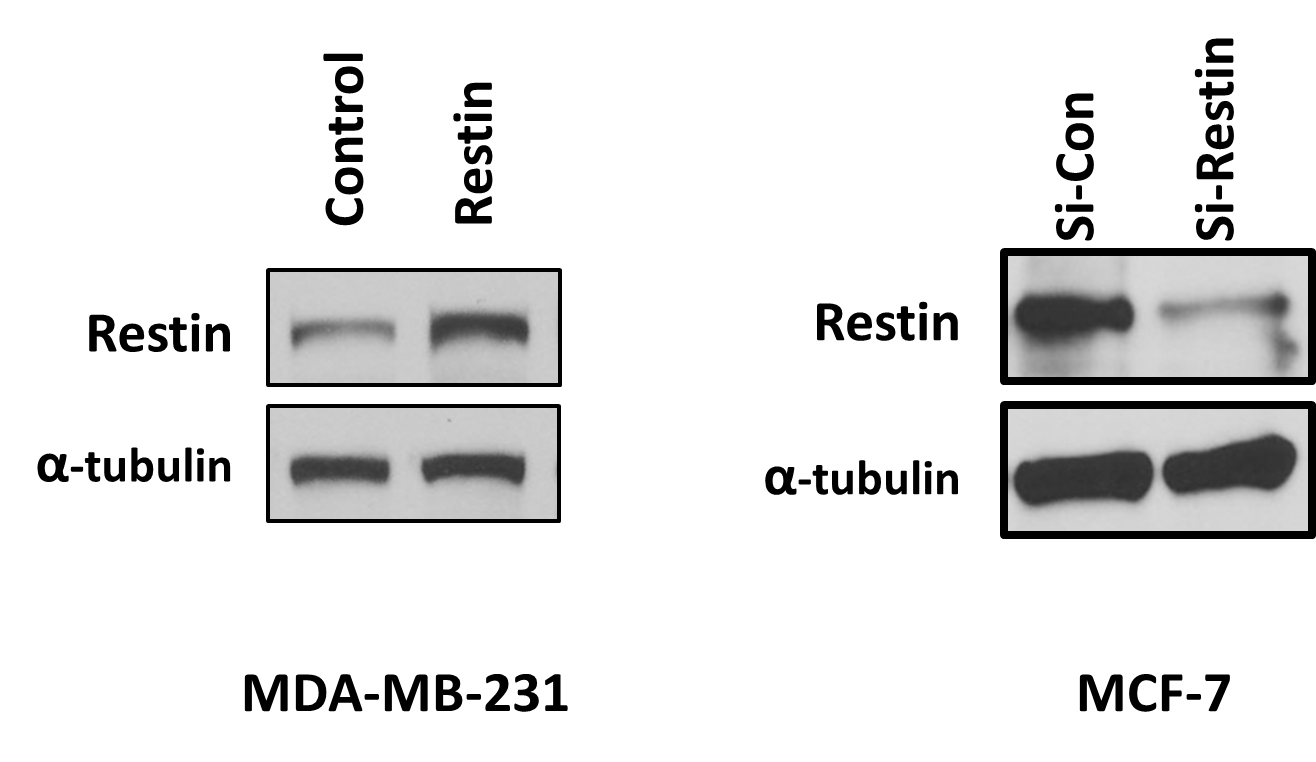
**

**Supplementary Figure 1.** Western blot was performed to confirm Restin expression levels in Restin overexpressed MDA-MB-231 cells and Restin knockdown MCF-7 cells. 2×10^5^ MDA-MB-231 and MCF-7 cells were seeded onto 6-well plates and transduced with 10 μl Control and Restin (Restin overexpression) or si-Con and si-Restin (Restin knockdown) lentivirus. 24 h later, transfected cells were trypsinized and reseeded onto 10-cm culture plates. Results presented here were representatives of three different experiments.


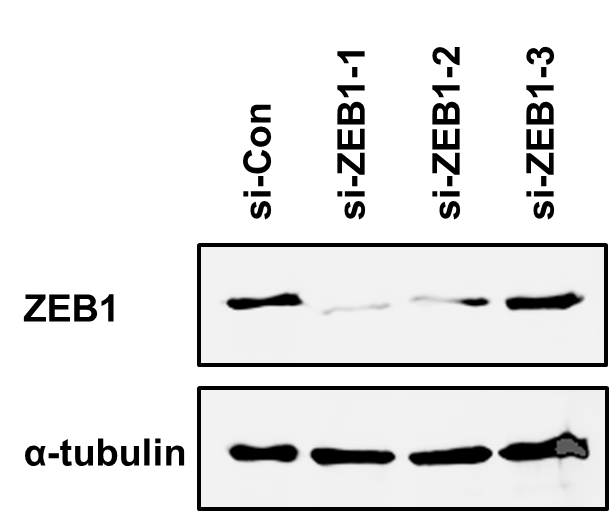


**Supplementary Figure 2.** Western blot was performed to detect ZEB1 expression levels in cells transfected with negative control and ZEB1 siRNAs. Result presented here was a representative of three different experiments.


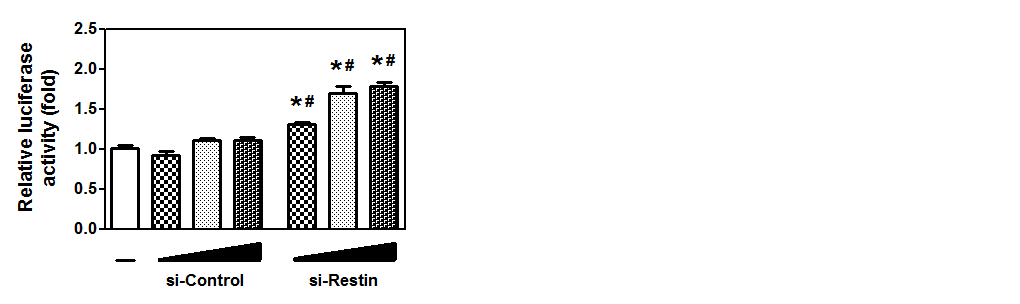


**Supplementary Figure 3.** ZEB1 3’UTR activity was determined in HEK293 cells by luciferase reporter assay upon Restin knockdown. HEK293 cells were seeded onto 24-well plates and transfected with ZEB1 3’UTR plasmids and different dose of Restin knockdown lentivirus (si-Restin). The firefly luciferase activity was normalized to that of the renilla luciferase. Values were expressed as means ± S.M. of at least three independent experiments. * *p* < 0.05 relative to cells without addition of lentivirus (-), ^#^ *p* < 0.05 relative to si-Control group.

**Supplementary Figure 4.** mir-200c and mir-141 levels were determined in Control and Restin overexpressed MDA-MB-231 cells by real-time PCR. Values were expressed as means ± S.M. of at least three independent experiments.

**Supplementary Figure 5.** mir-200c/141 promoter activity was determined by luciferase reporter assay. HEK293 cells were seeded onto 24-well plates and transfected with Restin overexpression lentivirus and mir-200c/141 promoter plasmids. The firefly luciferase activity was normalized to that of the renilla luciferase. Values were expressed as means ± S.M. of at least three independent experiments.


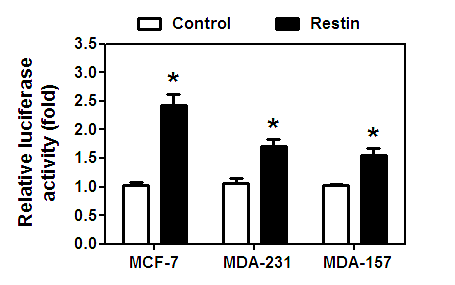


**Supplementary Figure 6.** mir-200b/a/429 promoter activities were measured by luciferase reporter assay in multiple cell lines. Restin overexpression lentiviruses were added into MCF-7, MDA-MB-231 and MDA-MB-157 cells. mir-200b/a/429 promoter plasmids were transiently transfected one day later. Luciferase activity was measured 48 h posttransfection. * *p* < 0.05 relative to Control lentivirus.


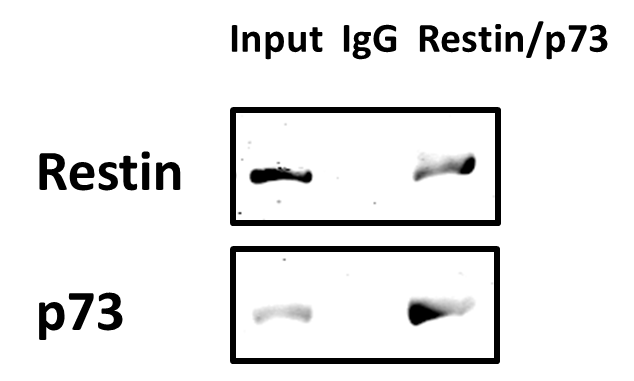


**Supplementary Figure 7.** Co-immunoprecipitation assay was performed to detect the endogenous interaction between Restin and p73. (Upper panel) MCF-7 cell extracts were immunoprecipitated with mouse IgG or anti-p73 antibody and then blotted with anti-Restin antibody. (Lower panel) Cell extracts were immunoprecipitated with mouse IgG or anti-Restin antibody and then blotted with anti-p73 antibody. Input, total cell lysates. Data were representative of three independent experiments.


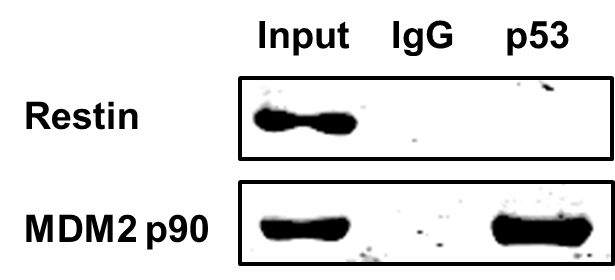


**Supplementary Figure 8.** Co-immunoprecipitation assay was performed to detect the endogenous interaction between Restin and p53. MCF-7 cell extracts were immunoprecipitated with mouse IgG or anti-p53 antibody and then blotted with anti-Restin (upper panel) and anti-MDM2 (C-18) antibodies (lower panel). MDM2 p90 is a positive control. Input, total cell lysates. Data were representative of three independent experiments.


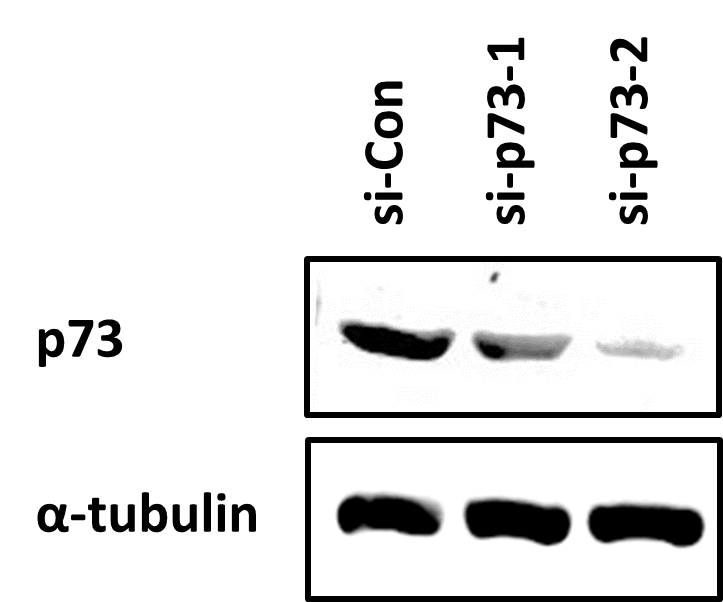


**Supplementary Figure 9.** Western blot was performed to detect p73 expression levels in cells transfected with negative control and p73 siRNAs. Result presented here was a representative of three different experiments.
